# Patients with emm1/T1 serotype invasive group A streptococci infections demonstrated more renal failure than patients with other serotypes: perhaps we should consider some confounders

**DOI:** 10.1186/s13054-020-03180-2

**Published:** 2020-07-29

**Authors:** Patrick M. Honore, Leonel Barreto Gutierrez, Luc Kugener, Sebastien Redant, Rachid Attou, Andrea Gallerani, David De Bels

**Affiliations:** ICU Department, Centre Hospitalier Universitaire Brugmann-Brugmann University Hospital, Place Van Gehuchtenplein, 4, 1020 Brussels, Belgium

We read with great interest the article by Björck et al. who concluded that in their study of critically ill patients with invasive group A streptococcal (iGAS) infections, emm1/T1 was the most dominant serotype and that patients with that serotype demonstrated more circulatory and renal failure than patients with other serotypes of iGAS [1]. We would like to make some comments. Intravenous immunoglobulins (IVIGs) are often used as a part of the treatment of iGAS [1]. We noted that 52% of the emm1/T1 serotype patients received IVIGs as compared to 28% of the patients with other serotypes [1]. The incidence of acute kidney injury (AKI) with IVIGs stabilized with glucose, maltose, d-sorbitol, mannitol, glycine, or l-proline has been found to be lower than that with sucrose-stabilized products [2]. AKI induced by sucrose-containing IVIGs is likely related to the toxic action of sucrose on the nephron, whereby excess sucrose in the kidney causes osmotic nephrosis [2, 3]. Whilst osmotic nephrosis has been reported with sucrose-free IVIGs, the incidence is much lower because the levels of these agents can be closely regulated by enzymes within the kidney [2, 4]. Similarly to sucrose, excessive glucose accumulation can have deleterious effects on the proximal tubules [5] and, since intravenous glucose infusion is known to produce a rapid increase in blood glucose and insulin levels in normal subjects, diabetic patients are at particular risk of AKI following administration of glucose-stabilized IVIGs [2]. The incidence of diabetes mellitus is not reported in the paper of Björck et al. [1]. It is possible that the increase of AKI in the emm1/T1 serotype group was due to IVIGs. It would be very interesting to know if the IVIGs given to patients in this study were sucrose-stabilized.

## Data Availability

Not applicable.

## Abbreviations

iGAS: Invasive group A streptococci
IVIGs: Intravenous immunoglobulins
AKI: Acute kidney injury
